# Cambridge University Medical Society

**Published:** 1890-12-13

**Authors:** 


### CAMBRIDGE UNIVERSITY MEDICAL SOCIETY.

This society held a meeting on Friday, December 5th, in the Anatomical Museum. After the private business had been settled, papers were read and discussion followed. Mr. Wherry read a paper on "Intra-cranial Aneurysm, consequent on Fracture of the Base of the Skull." The case presented many interesting features. A boy, aged 13, had been crushed by a truck, and was admitted to the Addenbrooke's Hospital on October 6th. A small quantity of blood exuded from his ears; he also vomited blood; he was very refractive, and could with difficulty be induced to keep his bed. Soon, however, he became better, and was steadily improving until, on October 28th, he had a severe pain in his neck and the occipital region of the skull. All the symptoms suggested tetanus, which, however, was not the case. There was considerable proctosis of the left eye. Leeches were applied to the left temple, and considerably alleviated the pain. Calomel was also given. On the 30th he became easier, and was quieter. On November 6th the general improvement was very great; there was then no proctosis of the eye or dilatation of the pupils. On the 16th he was suddenly seized with an acute pain in the orbital region. An incision was made to let out any fluid, and this greatly relieved the pain. Since then the patient has gradually been improving. With a wooden stethoscope it is possible to hear most distinct "bruits." Pressure on the right common carotid does not cause these to cease; but pressure on the left common carotid prevents the bruit. Accordingly, the question rather arises as to the advisability of tying the left carotid. Some discussion then followed as to whether it was an aneurysm, or a communication between an artery and a vein. Dr. Hill (Downing) and Mr. Douty (King's) regarded it as an aneurysm. Mr. Douty then showed some specimens of diseases of the eye, mounted by a new method. These were: (1) Melanotic sarcoma extending through the sclerotic into the optic nerve; (2) Detachment of the retina; (3) Glaucomatous cupping of the optic disc; (4) Glioma of the retina. The specimens were well prepared, and mounted, being covered by a convex magnifying lens, of about two diameters. Dr. Vauxhall also exhibited a specimen of ovary and Fallopian tubes, with a blood cyst in the ovary.

